# Quality Properties of Bakery Products and Pasta Containing Spent Coffee Grounds (SCGs): A Review

**DOI:** 10.3390/foods13223576

**Published:** 2024-11-08

**Authors:** Mitra Ahanchi, Elizabeth Christie Sugianto, Amy Chau, Ali Khoddami

**Affiliations:** 1 Faculty of Management, University of Warsaw, ul. Szturmowa 1/3, 02-678 Warszawa, Poland; 2 School of Life and Environmental Sciences, Faculty of Science, The University of Sydney, Sydney, NSW 2006, Australia

**Keywords:** coffee, spent coffee grounds, bioactive components, quality properties, bakery products

## Abstract

Coffee is one of the most consumed and popular beverages worldwide, and it produces a significant quantity of waste. Spent coffee grounds (SCGs) are one of the major waste products that can be used as an ingredient for creating novel foods. Therefore, the effect of incorporating varying percentages of spent coffee grounds (SCGs) on the quality properties of bakery products and pasta is reviewed. Chemically, SCGs alter protein, fat, fiber, ash, and bioactive compound levels in bakery and pasta products, improving nutritional value and promoting health benefits. The impact of SCGs on the physical characteristics of baked goods depends on factors like SCG concentration and processing methods, which influence product texture and structure. Sensory properties are vital for consumer acceptance. SCGs can add unique flavors and colors to baked goods, but more attention is needed to optimize the SCGs’ incorporation concentration for a better consumer appeal. In conclusion, integrating SCGs into bakery products and pasta offers nutritional enhancement, sustainability, and sensory improvement opportunities. Optimizing product quality allows manufacturers to leverage SCGs’ potential in the food industry.

## 1. Introduction

Coffee stands out as one of the most widely consumed and favored beverages globally, the second most traded commodity globally, just after petroleum, highlighting its significant influence and dominance in the global market [1,2,3]. The latest data from the International Coffee Organization (ICO) reveal that global coffee consumption reached 170.3 million 60 kg bags in 2021/2022 [4]. While coffee has energizing and uplifting benefits, it is crucial to consider the waste produced [5]. The amount of waste the coffee industry creates is anticipated to rise in the coming years due to the steady growth in coffee production and consumption [2]. The waste includes husks, pulp, mucilage, silverskins, and SCGs, which are produced on the farm and finished in the coffee shops [3]. SCGs are the primary waste material produced during processing, accounting for 0.6 tons per ton of coffee beans [1,6,7]. To adhere to current national regulations, waste management programs must be effective. The SCGs produced are often gathered by specialist organizations and sold for various uses, such as gardening, composting, bioenergy production, and mushroom cultivation [7].

There is much evidence of coffee’s health-promoting properties, which include anti-allergic, anti-inflammatory, anti-diabetic, anti-mutagenic, anti-cancer, antioxidant, and antimicrobial properties [2,8,9,10,11]. In addition, there is an inverse relationship with several illnesses, including Parkinson’s, Alzheimer’s, depression, fatty liver, and metabolic disorders [6,9,12]. Protective effects on various nervous, skeletal, reproductive, immune, and cardiovascular systems have also been reported [9,12]. The rich phytochemistry of coffee and its by-products, which comprise elements like caffeine and other physiologically active components primarily from the polyphenol and alkaloid groups, is responsible for these health advantages [2,6,8,9,12].

A key initiative in enhancing nutrition is raising the production of widely consumed items with substantial nutritional and biological significance. Proper nutrition plays an important role in treating and preventing many diseases [13]. SCGs are a source of macro-nutrients (carbohydrates, insoluble fiber, lipids, and protein), micro-nutrients (magnesium, sodium, potassium, and iron), and bioactive compounds (tannins, caffeine, and polyphenols) [5,6]. The upcycling of SCGs supports the development of functional foods and helps create a circular economy by repurposing waste materials. However, the impact of reducing environmental pollution may be limited for a few reasons. First, the global consumption of coffee results in a massive volume of SCGs, and while their application in food production can mitigate some waste, the scale at which SCGs can be incorporated into food products is relatively small compared to the total waste generated. Furthermore, there are challenges related to the sensory qualities (e.g., taste and texture) of SCG-enriched products, which may limit consumer acceptance and market scalability. In addition, there is a concern regarding the potential contamination by mycotoxins produced by certain molds, particularly under improper storage conditions [14,15,16,17].

Nonetheless, integrating SCGs into food production aligns with sustainable waste management practices by promoting the reuse of by-products, reducing landfill waste, and potentially lowering the demand for virgin agricultural inputs. This is particularly significant as it offers a more environmentally friendly alternative to conventional waste disposal methods, such as incineration or landfilling, which contribute to greenhouse gas emissions. While it may not drastically lower overall pollution, this approach supports broader sustainability goals by contributing to resource efficiency and waste minimization [6,7,11,18,19].

Bakery products are widely consumed and accepted [20,21], appealing to a broad range of age groups due to their widespread availability, convenience, and universal enjoyment [20]. Also, they are an essential source of nutrients (carbohydrates, proteins, vitamins, and minerals) [22,23,24]. However, these products have come into several issues in today’s world. Firstly, bakery products contain excessive levels of sugar and fat, which can lead to weight gain as well as the development of non-communicable diseases like diabetes, cardiovascular disease, and obesity [25]. The second issue is product deterioration, encompassing chemical, physical, and microbiological changes in baked goods [25,26].

Since SCGs contain bioactive compounds, including antioxidant dietary fibers and phenolic compounds, it can be expected that using this material in the formulation of bakery and pasta products assists in overcoming these issues [7,27]. On the other hand, using SCGs in baking products has been shown to improve the sensory, physical, and chemical properties of these products [18,28,29,30,31,32].

The innovation of this review lies in the comprehensive evaluation of SCGs as a sustainable food ingredient. While previous studies have explored various aspects of SCG utilization, this review focuses on their specific impact on bakery products and pasta, addressing gaps in the existing literature by integrating findings from different fields. This review is new in compiling and presenting past research data regarding the effects of incorporating SCGs into the chemical, physical, microbiological, and sensory properties of bakery products. Moreover, it promotes the environmental benefits of incorporating SCGs into food products, such as reducing food waste and promoting circular economy practices. By exploring these dual benefits, we aim to contribute to the growing body of research that seeks sustainable solutions within the food industry.

## 2. Methods of Review

A comprehensive search was conducted using the following reputable databases: Google Scholar, ScienceDirect, PubMed, Springer, and Wiley, to find related peer-reviewed published research. Studies were selected using the keywords “spent coffee grounds” (SCGs), “grain-based products”, “bakery products”, “bread”, “cakes”, “biscuits”, “cookies”, “wafers”, “muffins”, “crackers”, and “pasta”. They were thoroughly read to ensure that the chosen papers addressed the research topic. The authors, the year of publication, the goals, the methodology, and the findings were among the data assessed. Compatibility with the primary topic and readability were the two requirements for inclusion. The exclusion criteria included animal studies, literature reviews without full text, and incompatibility with the core theme.

After searching the databases for the literature, classifying and analyzing the outcomes, and investigating the studies, 21 studies were found to integrate this review. Then, the papers’ full texts were examined to ensure they addressed the topic of the current investigation. The chosen literature was published from 2012 onward.

## 3. Results and Discussion

Out of the 21 works of literature that were assessed, 47.61% of the studies produced cookies (n = 10), 14.28% made bread (n = 3), 14.28% made biscuits (n = 3), 9.52% (n = 2) made cakes, 9.52% (n = 2) made muffins, and 4.76% (n = 1) made pasta that contained SCGs. Among these studies (n = 21), 95.23% conducted chemical analyses (n = 20), 80.95% conducted physical analyses (n = 17), 19.04% conducted microbial analyses (n = 4), and 76.19% conducted sensory analyses (n = 16).

The research that examined bakery products and pasta prepared with SCGs is displayed in Table 1. A massive variation was seen in the amount of SCGs incorporated into baked products. Studies involving cookies found a minimum of 0.05% and a maximum of 30.0% SCGs incorporation. The amounts for breads and biscuits ranged from 2.0% to 11.0%, while the contents for pasta and cakes varied from 1.0% to 10.0%. Muffins showed the greatest variation in SCGs incorporation, with the minimum amount being 1.0% and the maximum being 61.0%. Of the studies, 66.66% (n = 14) also mentioned the variety of coffee they had used: ‘Arabica’ and/or ‘Robusta’.

Table 1 is instructed to precisely present the impact of the inclusion of SCGs or SCG derivatives on the chemical, physical, and sensory properties of baked products.

### 3.1. Bakery Products’ Chemical Properties

#### 3.1.1. Cookies

In terms of moisture content, Aguilar-Raymundo et al. [27] noted that cookies containing 10.0%, 17.5%, and 25.0% SCGs demonstrated a reduced moisture content compared to the control cookies that did not include SCGs. The decrease in moisture levels may be attributed to the elevated fiber content present in SCGs, which can absorb water. This phenomenon could contribute to an enhanced shelf life of cookies by lowering their water activity. Similarly, Oliviera Batista et al. [32] reported a decrease in moisture in gluten-free cookies due to the inclusion of SCGs. Azuan et al. [29] presented divergent results, indicating that cookies supplemented with SCG extract from Arabica coffee beans exhibited a higher moisture content. This suggests that the impact of SCGs on moisture may depend on the type of coffee bean and processing method. Desai et al. [33] also observed that adding roasted green spent coffee grounds flour (RGCSF) and unroasted green spent coffee grounds flour (UGCSF) to cookies increases the moisture content of this product. Trà et al. [18] conducted a study on cookies incorporating SCGs derived from Robusta coffee beans, revealing a marginal rise in moisture content. However, the variations noted were minimal compared to the findings reported by Azuan et al. [29].

Regarding lipid stability, Koay et al. [7] found that adding SCG powder to shortbread improved lipid stability due to its high dietary fiber and antioxidant properties. The findings indicate that incorporating SCGs resulted in an enhancement of both, which has the potential to enhance the nutritional value of baked goods, in addition to prolonging their shelf life through lipid stabilization. Meerasri et al. [34] similarly illustrated that substituting butter with oil derived from SCGs in cookie formulations enhanced the total phenolic content (TPC) and antioxidant activity while preserving moisture levels. This suggests that SCG oil is a viable alternative to butter, offering enhanced lipid stability. Also, Sérioa et al. [35] investigated the antioxidant activity of cookies containing SCG extracts with 1.0%, 2.0%, and 3.0% concentrations. They demonstrated that the increase in the amount of SCG extract enhanced the antioxidant activities of cookies. SCGs contain a variety of bioactive compounds, including phenolics, chlorogenic acid, and caffeine, which play a significant role in their antioxidant capabilities. Sharma et al. [36] investigated the impact of roasted spent coffee grounds (RSCGs) and spent coffee ground residue (SCGR) on the composition of cookies, revealing that RSCGs provided notably greater concentrations of bioactive compounds in comparison to SCGR. Roasting SCGs appears to increase the accessibility of phenolic compounds and various antioxidants, thereby enhancing lipid stability and prolonging the shelf life of the goods.

In terms of nutritional quality, Aguilar-Raymundo et al. [27] observed that cookies fortified with SCGs demonstrated elevated concentrations of fat, crude fiber, and ash in comparison to the control cookies, whereas the protein content was diminished. The enhancement of fiber content, especially in the form of crude fiber, represents a significant nutritional advantage of cookies enriched with spent coffee grounds (SCGs). Fiber is crucial for supporting gastrointestinal health and may decrease the risk of chronic conditions, including cardiovascular disease and obesity [37,38]. Azuan et al. [29] also observed improvements in ash, fat, and protein content when extracts from SCGs were incorporated into cookies. Desai et al. [33] conducted a comparative analysis of the nutritional effects of roasted and unroasted spent coffee grounds incorporated into cookies. Their findings indicated that RGCSF significantly increased the protein, ash, and soluble dietary fiber levels, whereas UGCSF was more effective in enhancing the insoluble dietary fiber content. This suggests that roasting may alter the fiber profile and nutrient retention in SCGs, making roasted SCGs a more suitable choice for increasing protein and soluble fiber in cookies. Oliveira Batista et al. [32] conducted a study on the incorporation of SCGs into gluten-free cookies, revealing that elevated levels of SCGs corresponded with enhanced concentrations of protein, lipids, ash, and fiber. The findings align with Aguilar-Raymundo et al. [27] and Azuan et al. [29], who documented an increase in fiber and ash content in cookies supplemented with SCGs. Koay et al. [7] further illustrated that the incorporation of SCG powder into shortbread resulted in a decrease in the caloric value of the cookies while simultaneously enhancing their nutritional characteristics. The elevated dietary fiber content of SCGs contributed to a reduction in the glycemic index of the baked products. In addition, Castaldo et al. [39] reported higher antioxidant activity in cookies enriched with 5.0% SCGs, confirming the antioxidant benefits of SCGs.

The studies consistently show that SCGs, whether in extract, flour, or powder form, significantly improve cookies’ antioxidant activity, TPC, and fiber levels [33,34,35,39]. There is a general trend of increased ash, fat, and protein content with SCGs inclusion, with variations in moisture content depending on the SCGs processing method and bean type [27,29,32]. The roasting of SCGs enhances bioactive compounds and antioxidants, while SCG oil can effectively replace butter in cookie formulations [33,34,36]. Overall, SCGs’ high fiber content and antioxidant properties make them a valuable functional ingredient for improving the nutritional profile of cookies, potentially extending shelf life by reducing moisture and enhancing health benefits, such as improved gastrointestinal health, reduced risk of cardiovascular diseases, and weight management [37,38,40]. The findings indicate that SCGs offer versatile benefits, particularly in gluten-free and low-calorie cookie formulations [7,32,41].

#### 3.1.2. Breads

Daniel et al. [42] assessed the effect of adding SCGs at concentrations of 2.0%, 4.0%, 6.0%, 8.0%, and 10.0% on the chemical features of bread. They observed that bread with 10.0% SCGs had the highest protein level. Fat content varied among samples containing coffee grounds, possibly influenced by the amount of oil used. Also, the coffee brewing process affects the fat content because a considerable portion of the unsaponifiable fat compounds is eliminated during this process [43]. Bread with 10.0% SCGs had the highest ash content, which indicates a higher amount of inorganic elements in SCGs than regular white wheat flour. The fiber level of all breads containing SCGs was also noticeably enhanced compared to white bread, with the highest level found for the sample containing 10.0% SCGs. Total carbohydrates were higher in samples with 2.0% and 4.0% SCGs, suggesting that SCGs-enriched bread could be a beneficial energy source. SCG-supplemented breads also showed higher levels of total phenolics and flavonoids, likely due to the high content of these compounds in SCGs [42].

Chau [44] examined the chemical properties of white bread formulated with 2.0% and 4.0% SCG varieties of Robusta and Arabica (data are not published). The study found that adding both varieties of SCGs into bread enhanced the level of resistant starch and reduced digestible starch content. Both types of SCGs enhanced the total phenolic value and antioxidant capacity in breads. Bread with 2.0% Robusta SCGs had a slightly higher phenolic content and antioxidant capacity than bread with 2.0% Arabica SCGs, while 4.0% Robusta SCGs bread showed significantly higher values than 4.0% Arabica SCGs bread. Differences in phenolic content may be attributed to SCGs source and coffee extraction parameter variations [45]. The study suggests that SCGs could serve as a potential antioxidant source in bread, potentially protecting against oxidative damage and inhibiting starch-digesting enzymes [42].

Both studies provide valuable insights into the chemical properties of SCG-enriched bread, demonstrating improvements in protein, fiber, mineral content, phenolic compounds, and antioxidant capacity [42,44]. SCGs show promise as a functional ingredient, offering potential health benefits such as improved gut health, better glycemic control, and enhanced antioxidant activity [37,38,42]. However, further studies are needed to explore the sensory and consumer acceptability aspects, as well as the long-term health impacts of incorporating SCGs into bakery products. The variations in the chemical composition of bread due to differences in SCGs concentration and source emphasize the need for careful optimization when formulating SCG-enriched bread for commercial purposes [42,44].

#### 3.1.3. Biscuits

Martinez Saez et al. [30] compared the level of amino acids in biscuits formulated with 3.50%, 3.64%, 3.77%, 3.94%, 4.24%, and 4.40% of SCGs from Robusta coffee beans with commercial biscuits. They reported a significant difference between biscuits containing 4.24% and 4.40% SCGs and commercial biscuits. The level of amino acids in commercial biscuits was significantly lower than in biscuits containing 4.24% and 4.40% coffee grounds. This finding suggests that the inclusion of SCGs at these concentrations enhances the nutritional profile of biscuits by increasing their amino acid content, which is essential for improving the nutritional value, particularly for protein quality. This could be linked to the bioactive compounds and residual proteins in SCGs, which enhance the overall protein quality in biscuits and could be valuable for creating more nutrient-dense bakery products.

Ali et al. [46] investigated the effect of SCGs with levels of 2.0%, 4.0%, and 6.0% inclusion on the chemical composition of biscuits. Their results indicated that although the inclusion of SCGs reduced protein content, other beneficial changes were observed. Specifically, the fiber, ash, and moisture content increased with higher concentrations of SCGs, enhancing the biscuits’ overall nutritional value, particularly the dietary fiber, which plays a critical role in digestive health. The absence of significant differences in fat content suggests that SCGs do not substantially alter the lipid profile of biscuits, which could be an advantage for maintaining a consistent texture and mouthfeel. This study highlights that while there is a trade-off with reduced protein content, the increased fiber and mineral content from SCGs may offer functional and health benefits that make these biscuits appealing from a nutritional standpoint.

Campos-Vega. [47] evaluated the chemical composition of biscuits containing 7.77% SCGs (added with fructooligosaccharides; SCG-FOS) and 11.11% SCGs antioxidant dietary fiber (SCF). Their findings revealed that the SCF biscuits exhibited the highest protein levels, while the SCG-FOS biscuits had the highest lipid content. Notably, the SCF biscuits also had the most total dietary fiber, suggesting that SCGs, especially in combination with other dietary fiber sources, significantly improve the fiber content of biscuits. However, while the total phenolic content remained similar across all biscuits, the extractable antioxidant capacity varied, with traditional biscuits (TBs) exhibiting the highest antioxidant capacity. This discrepancy could be attributed to differences in the bioavailability of phenolic compounds in SCGs and their interactions with other biscuit ingredients. Interestingly, despite the enhanced nutritional properties, no significant differences in the carbohydrate content were noted, indicating that incorporating SCGs and other functional ingredients does not drastically alter the energy content of the biscuits. This study highlights the potential for SCGs to enhance specific nutritional attributes, such as protein and fiber content. However, further optimization is required to retain or boost the antioxidant capacity.

In summary, the inclusion of SCGs in biscuits presents both nutritional benefits and certain limitations [30,46,47]. Studies have shown that while SCGs can enhance dietary fiber and mineral content, they may reduce protein content, depending on the formulation [46,47]. Furthermore, the antioxidant capacity of SCG-enriched biscuits may vary, possibly due to the interactions between SCGs and other ingredients [47]. Overall, SCGs offer potential as a functional ingredient in biscuits, contributing to enhanced fiber and nutrient density [46,47]. However, further research is necessary to optimize formulations for improved antioxidant properties and protein retention.

#### 3.1.4. Cakes

The protein, fat, dietary fiber, and ash levels of sponge cakes containing SCGs (2.0%, 4.0%, and 6.0%) were studied by Hussein et al. [31]. Their results revealed that adding SCGs did not significantly change the protein and fat content compared to the control sample, suggesting that SCGs do not interfere with the stability of protein or fat in baked products. However, a notable rise in the dietary fiber and ash content was observed as the SCG concentration increased. This suggests that SCGs can enhance the fiber content of cakes, improving their nutritional profile for consumers looking for fiber-enriched products. The increase in ash indicates a higher mineral content in the cakes, which could provide additional health benefits.

Similarly, Ahmad et al. [28] investigated the impact of incorporating SCGs (1.0%, 2.0%, and 3.0%) into sponge cakes. Their study emphasized the potential functional and nutritional improvements that SCGs offer. While the control sponge cake had the highest dry matter value, cakes enriched with SCGs showed increased ash, crude fiber, and minerals levels, especially at the 3.0% concentration. These results indicate that SCGs can enhance the mineral and fiber content in cakes, offering a more nutrient-dense product. Moreover, SCG-enriched cakes also demonstrated elevated levels of total phenolics, tannins, and antioxidant activity, highlighting SCGs’ ability to impart bioactive compounds into the product. The higher antioxidant activity could be particularly appealing from a health perspective, as antioxidants play a crucial role in neutralizing free radicals and may contribute to reducing the risk of chronic diseases.

These studies collectively suggest that SCGs can be a valuable ingredient in cakes, maintaining their elemental macronutrient composition (e.g., protein and fat) and enhancing their functional and nutritional properties, especially regarding fiber, minerals, and antioxidants. The use of SCGs could cater to health-conscious consumers seeking cakes with added health benefits, particularly in terms of dietary fiber intake and antioxidant capacity [28,31].

#### 3.1.5. Muffins

Severini et al. [48] evaluated the chemical properties of muffins supplemented with 15.0% and 30.0% SCGs from Arabica coffee beans. They observed that adding SCGs to muffins increased the antioxidant capacity, phenolic compounds, and dietary fiber. The authors suggest that SCGs supplementation could serve as a functional ingredient, providing added health benefits.

Benincá et al. [49] examined the antioxidant properties of muffins supplemented with 1.0%, 16.0%, 31.0%, 46.0%, and 61.0% of SCGs from Arabica coffee. They reported that adding SCGs increased antioxidant activity, total phenolics, caffeine, chlorogenic acid (5-CGA), and trigonelline contents. These bioactive compounds are well known for their potential health benefits, such as reducing oxidative stress and improving cardiovascular health. The dose-dependent relationship observed in this study emphasizes the potential of SCGs as a valuable addition to muffins.

These studies highlighted that incorporating SCGs into muffins is a promising strategy for enhancing the nutritional profile of bakery products. The increase in antioxidant capacity, phenolic compounds, and dietary fiber suggests that SCGs could be used to develop functional foods with added health benefits [48,49].

#### 3.1.6. Pasta

Sugianto [19] studied the moisture value of pasta containing 0.0%, 4.0%, 8.0%, and 10.0% SCGs from Arabica coffee beans. They did not observe any noticeable differences in the moisture value between the samples enriched with 4.0%, 8.0%, and 10.0% SCGs compared to the 0.0% SCGs pasta. One possible explanation for the lack of differences in moisture content could be the relatively small quantity of added SCGs. The fibrous nature of SCGs may also limit their interaction with water molecules compared to other ingredients. Furthermore, the drying temperature is crucial in regulating moisture loss, and it is possible that the SCG-enriched pasta behaved similarly to the control sample under the same drying conditions. This suggests that the drying process may have a more dominant effect on moisture content than the addition of SCGs. In addition to moisture content, Sugianto noted the potential impact of SCGs on non-enzymatic browning reactions. Moisture levels play a crucial role in browning processes, where a higher moisture content generally limits the extent of Maillard reactions and caramelization during drying.

This study provides valuable insight into the moisture behavior of SCG-enriched pasta. The results indicate that the inclusion of up to 10.0% SCGs does not significantly affect moisture retention during drying [19].

**Table 1:** The impact of SCGs or SCG’s derivative inclusion on chemical, physical, and sensory properties of baked products.

Product	SCGs or SCGs Derivatives Concentrations (%)	Chemical	Physical	Sensory	References
	10.0, 17.5, and 25.0	Increased fat, crude fiber, and ash. Decreased moisture and protein.	All the treatments were soft, crumbly, and lacked elasticity.	The 17.5% SCGs incorporation is preferred in terms of impact on hardness, granularity, flavor, and taste.	[27]
	10.0, 20.0, and 30.0	Increased protein, ash, lipid, and fiber contents. Decreased moisture content.	Improved spread ratio and specific volume. Led to higher mass loss at q 10% inclusion rate. Decreased hardness at a 20% and 30% inclusion rate. Color shifted to dark brown.	The 10% and 20% inclusions were acceptable in terms of flavor and general acceptance.	[32]
	0.27, 0.53, 0.80, 1.07, and 1.33	Increased moisture, ash, fat, protein, crude fiber, calories, antioxidant activity, and TPC.	Enhanced a* and decreased L* and b*. Significant impact with the inclusion of 0.80%. Enhanced fracturability.	The highest appearance scores were related to the 0% extract. The brightest score was related to the 0.53% extract.	[29]
	3.0, 6.0, 10.0, and 12.0	Increased water activity and moisture content with UGCSF and RGCSF.	Increased diameter and spread ratio with RGCSF. Reduced L*, a*, and b* with UGCSF and RGCSF.	The 10% and 12% were preferred in color, appearance, aroma, taste, crispiness, and general acceptability.	[33]
	0.05, 0.10, 0.15, 0.20, and 0.25	Increased moisture, fat, ash, total phenolic contents, and antioxidant activity. Decreased total carbohydrate and starch content.	Increased dough hardness. Reduced adhesiveness, cohesion, and springiness. Caused darker color.	Steady general consumer acceptance for concentration ranged from 0.05% to 0.20%.	[18]
Cookies/shortbread	2.0, 4.0, 6.0, 8.0, and 10.0	Increased calorie, moisture, ash, crude protein, crude fiber, total phenolic contents, and antioxidant activity. Decreased carbohydrate and peroxide values.	Reduced water loss. Reduced L* and b* values but enhanced the a* value. Increased hardness at lower concentrations.	Preferred 10% inclusion in terms of aroma and hardness.	[7]
	10.0, 20.0, and 30.0	Increased phenolic content and antioxidant activity.	Increased the diameter. Remained unchanged in the thickness.	Lower flavor rating scores for 20% and 30% inclusion. No significant differences in the attributes of color, hardness, crumbness, and odor scores.	[34]
	1.0, 2.0, and 3.0	Enhanced antioxidant activity.	-	Impact on color: preferred at lower levels.	[35]
	5.0, 7.0, and 10.0	Inclusion of RSCGs and SCGR, enhanced fiber level, total phenolics, and antioxidant activity.	The treatment with 7% RSCGs had a higher hardness, whereas the treatment with 10% SCGR had a higher hardness Both treatments had similar fracturability values. Enhanced a* and decreased b* color values.	The 7.0% SCGR treatment was preferred in terms of texture and taste.	[36]
	5.0	Enhanced antioxidant activity.	-	-	[39]
	2.0, 4.0, 6.0, 8.0, and 10.0	Increased protein, total phenolics, antioxidant activity, and total flavonoid levels. Highest ash, mineral, and fiber content for a 10% inclusion rate. Higher carbohydrate for 2.0 and 4.0% inclusion rates.	Reduced water loss. Higher loaf volume at a 2.0% inclusion rate.	Preferred color, aroma, texture, and general acceptability were for a 2.0% inclusion rate.	[42]
Bread	2.0 and 4.0	Enhanced resistant starch, total phenolics, and antioxidant activity	Darker and redder crumb and crust color.	-	[44]
	4.0	-	Decreased hardness, springiness, elasticity, and cohesiveness. Reduced resilience, brightness, redness, and yellowness. Heavier loaf and a lower specific volume.	Preferred in terms of aroma and taste.	[50]
	3.50, 3.64, 3.77, 3.94, 4.24, and 4.40	Increased amino acid contents.	No significant differences in texture.	Increased the color scores at 3.50%, 3.64%, and 4.24% inclusion. Similar overall acceptance for 3.50%, 3.64%, and 4.24% inclusion rates and control.	[30]
Biscuit	2.0, 4.0, and 6.0	Enhanced fiber, ash, and moisture content. Reduced protein content.	Reduced the L* and b* values, and Enhanced the a* value.	No significant difference in aroma and taste at 2.0% and 4.0% inclusion rates. No significant difference in texture and overall acceptance.	[46]
	7.77, and 11.11	The highest protein and fiber were related to the SCF, while the highest lipid was related to the SC-FOS. Total phenolic content was higher in SCF.	-	-	[47]
Sponge Cake	2.0, 4.0, and 6.0	Increased dietary fiber and ash contents.	Intensified browning in both the crust and crumb. Increased a* and b* values. Increased dough water absorption and development time. Decreased dough stability. Improved mixing tolerance index. Increased weight and specific volume. Improved baking quality. Increased adhesiveness. Decreased hardness, gumminess, and chewiness.	Color, taste, smell, texture, appearance, and general acceptability were preferred for 2.0% and 4.0% inclusion rates.	[31]
	1.0, 2.0, and 3.0	Reduced the dry matter. Enhanced crude fiber, all minerals (except Na), ash, total phenolics, total tannin contents, and antioxidant activity.	Decreased L* value of the crust and crumb. No notable change in a* value. Decreased b* value for both crust and crumb.	Negatively impacted the appearance, shell color, crumb color, texture, taste, and smell.	[28]
Muffins	5.25, and 10.5	Enhanced antioxidant activity, total phenolics, and total dietary fiber.	Reduced the specific gravity. Reduced the L*, a* and b* color values.	Received high sensory scores in terms of appearance, color, odor, taste, softness, and overall acceptance.	[48]
	1.0, 16.0, 31.0, 46.0, and 61.0	Increased the total phenolic content, antioxidant activity, caffeine, chloro-genic acid (5-CGA), and trigonelline.	Increased the L* value and hardness. Reduced the a* and b* color values. No significant difference in elasticity, chewiness, and cohesiveness across various concentrations.	-	[49]

### 3.2. Bakery Products’ Physical Properties

#### 3.2.1. Cookies

Aguilar-Raymundo et al. [27] studied the effect of adding SCGs at 10.0%, 17.5%, and 25.0% on the textural properties of cookies using a texture analyzer. Despite varying SCGs percentages, no significant differences were observed in the samples’ cohesiveness, hardness, and springiness. Hardness values ranged from 33 to 51 N, while cohesiveness and springiness remained relatively low, ranging from 0.08 to 0.11 and 11.9% to 19.4%, respectively. Overall, the cookies were characterized as soft, crumbly, and lacking elasticity.

Azuan et al. [29] evaluated cookies’ color and textural properties supplemented with 0.25%, 0.53%, 0.80%, 1.07%, and 1.33% SCG extracts. These researchers used the Konica Minolta color measuring system and the texture analyzer to check the color and texture properties of the cookies. The researchers observed changes in cookies’ color and textural properties when incorporating SCGs extracts. The increase in the amount of added SCG extract in cookies resulted in decreasing lightness (L*) and yellowness (b*) while redness (a*) increased. These color changes are attributed to the Maillard reaction during baking and the SCG extract’s original dark color [31,37]. Regarding hardness, there was no notable variation between the samples enriched with the SCG extract and the control sample. However, in terms of fracturability, there was a notable variation between the samples enriched with the SCG extract and the control sample. The samples’ fracturability rose when the SCG extract was incorporated; adding a 1.33% SCG extract gave the maximum fracturability value of 40.03 mm, while the control sample’s fracturability value was 38.43 mm [29].

Desai et al. [33] investigated the effect of adding RGCSF and UGCSF (3.0%, 6.0%, 10.0%, and 12.0%) on the physical features of cookies. These researchers used the colorimeter and the texture analyzer to check the color and texture attributes of the cookies. They indicated that cookies with 10.0% UGCSF were heavier than control cookies, likely due to the higher dietary fibers in UGCSF that retain moisture during baking. Conversely, cookies with 12.0% RGCSF were lighter than controls. RGCSF increased the cookie diameter and spread ratio, indicating desirable quality traits that consumers prefer. Cookies with 10.0% UGCs had the lowest weight and maximum spread ratio. Thickness varied, with cookies containing 10.0% and 12.0% UGCSF being the thickest, while those with 10.0% RGCSF were the thinnest. Increasing UGCSF and RGCSF concentrations led to reduced lightness (L*), redness (a*), and yellowness (b*) parameters, attributed to the darker RGCSF and caramelization of sugars in UGCSF during baking.

Meerasri and Sothornvit [34] studied the physical characteristics of cookies made with 10.0%, 20.0%, or 30.0% SCG oil. These researchers used dynamic oscillatory tests to determine the rheological features of dough. The diameter was measured as an average value when placing four cookies edge to edge. Thickness was measured using a vernier caliper by taking the average of stacking four cookies on top of each other. The spread ratio was calculated by dividing the average value of the diameter by the average value of the thickness of the cookies [51]. They also used a texture analyzer and a spectro-guide spectrophotometer to measure the texture and color characteristics. They exhibited that cookies made with 10.0%, 20.0%, and 30.0% SCG oil exhibited reduced storage modulus (G′) and loss modulus (G″) values as opposed to cookies made with butter. The addition of SCG oil weakened the dough microstructure and reduced the viscosity. The cookies’ diameters increased when using SCG oil, likely due to the weaker microstructure, allowing easier spreading during baking. However, the thickness and spread ratio remained unaffected by the SCG oil substitution. A substantial reduction in hardness was observed in cookies with higher SCG oil content, suggesting altered texture due to increased softening of the dough microstructure. In addition, the color parameters showed minimal differences, but when 30.0% SCG oil was used, the lightness (L*) and yellowness (b*) notably decreased [39].

In another study, Sharma et al. [36] examined the effect of 5.0%, 7.0%, and 10.0% RSCGs and SCGR on the texture and color of cookies using a texture analyzer and a LabScan XE spectrophotometer, respectively. The control treatment had a fracturability of 14.99 mm and a hardness of 4.74 N. Cookies with 7.0% RSCGs and 10.0% SCGR showed texture profiles similar to the control regarding hardness and fracturability. Cookies with 7.0% RSCGs had a hardness of 3.15 N and a fracturability of 13.23 mm, while those with 10.0% SCGR had a hardness of 4.80 N and a fracturability of 13.2 mm. SCG-enriched cookies appeared darker due to the presence of melanoidins, unlike SCGR cookies. RSCGs and SCGR-added cookies exhibited increased redness but decreased yellowness compared to the control.

Trà et al. [18] studied cookies containing SCGs (0.0%, 0.10%, 0.15%, 0.20%, and 0.25%). They used a texture analyzer and Konica Minolta color system to evaluate texture and color features. These researchers observed that the hardness of cookie dough increased with higher concentrations of SCGs in the formulation. At the same time, adhesiveness, cohesion, and springiness decreased marginally. Higher SCGs concentrations reduced cookie brightness and increased ΔE values, indicating a darker color and reduced red and yellow tints.

Oliviera Batista et al. [32] assessed the physical characteristics of gluten-free cookies enriched with SCG concentrations of 10.0%, 20.0%, and 30.0%. The spreadability was determined using the ratio between the diameter and thickness values of the cookies after baking. The specific volume was expressed using the apparent volume (millet seed displacement method) and cookie mass after baking. The hardness of the gluten-free cookies was obtained using a TA.XT2 texture analyzer. Findings showed that incorporating 10.0% or 20.0% SCGs into gluten-free cookies improved their physical characteristics, including a better spread ratio and specific volume, compared to the control. However, the cookies with 10.0% SCGs exhibited a higher mass loss, while 30.0% SCGs did not affect these metrics. The higher fiber content in 20.0% and 30.0% SCGs cookies may have competed for water during baking, reducing the water availability for sugar dissolution and increasing viscosity, thereby decreasing the spread ratio [33,52]. SCGs’ higher lipid level and larger particle size may have also contributed to the increased spread ratio [52]. Hardness remained unchanged at 10.0% SCGs but decreased at 20.0% and 30.0% SCGs [30], likely due to the enhanced water retention and softer texture from high fiber and lipid levels [27]. Moreover, items with higher lipid levels might have resulted in a softer texture [51]. SCGs improved textural characteristics, potentially making gluten-free cookies more robust than regular cookies [53]. In terms of color, the gluten-free cookies without SCGs were lighter in color, whereas the gluten-free cookies incorporated with SCGs presented a dark brown color [32]. These color shifts could be related to the dark hue of SCGs and the sugars’ caramelization during baking [37].

Koay et al. [7] investigated the effect of 2.0%, 4.0%, 6.0%, 8.0%, and 10.0% SCGs on shortbread dimensions, color, and texture. Diameter and thickness were measured with a digital vernier caliper, and the spread ratio was calculated by dividing the diameter by the thickness. Additionally, the Konica Minolta color measuring system and the texture analyzer were applied to investigate the color and texture attributes of the cookies. They found noticeable changes in diameter and thickness before and after baking. These changes were consistent across samples with different SCGs incorporation levels. However, there was a significant reduction in water loss in SCG-containing samples, likely due to the water-retaining properties of SCGs fibers. Despite minimal differences in weight and spread, baking caused a slight decrease in both parameters for all samples due to moisture loss [37]. Regarding color, SCGs incorporation resulted in darker shortbread with lower brightness (L*) and yellowness (b*) values, attributed to SCG’s dark color and enzymatic browning during baking. However, the redness (a*) value increased with higher SCGs levels, likely due to non-enzymatic browning reactions during baking. Texturally, the hardness of shortbread varied with the SCGs content. While the hardness initially increased with low SCGs levels, it gradually decreased with higher SCGs contents. This was attributed to the substitution of flour with SCGs. Higher SCG contents increased moisture in the shortbread, leading to a softer texture over time [7].

#### 3.2.2. Breads

Daniel et al. [42] evaluated the impact of adding SCGs in concentrations of 2.0%, 4.0%, 6.0%, 8.0%, and 10.0% on the physical features of bread. The highest loaf volume was associated with the sample containing 2.0% SCGs. Loaf volume was measured 50 min. after the loaves were removed from the oven using the rapeseed displacement method. The baking loss is obtained by subtracting the loaf weight (g) from the initial dough weight. In terms of baking loss, bread baked with a 10.0% SCGs concentration lost less water (9.29%), resulting in a higher weight for the bread. This could be due to fiber, a component of SCGs, having a high water retention capacity.

Rwubatse et al. [50] studied the physical attributes of whole wheat bread formulated with 4.0% SCGs. These researchers used a texture analyzer and a color reader to evaluate texture and color properties. Additionally, the specific volume and density were determined by an AACCI-approved technique 10-05-01 [54]. They stated that bread enriched with SCGs exhibited lower hardness compared to the control bread, indicating a softer texture. Prolonged dough fermentation combined with SCGs led to decreased springiness, or the bread’s ability to rebound after compression, suggesting reduced elasticity [55]. Moreover, the addition of SCGs decreased cohesiveness, making the bread more prone to breaking or crumbling. Combining SCGs with whole wheat flour further reduced the resilience associated with bread staleness [50]. SCG-enriched bread also displayed lower values of lightness (L*), redness (a*), and yellowness (b*) compared to the control, attributed to SCG’s antioxidant properties mitigating non-enzymatic browning reactions [30]. SCG-enriched bread was heavier than the control, especially when fermented for extended periods, and had a lower specific volume due to SCGs fiber decreasing the gluten network essential for gas retention [50,56]. Furthermore, SCGs incorporation decreased bread density by enhancing dietary fiber content. Breads fermented for different durations exhibited variations in density, with SCG-enriched bread from shorter fermentation periods being denser compared to those from longer fermentation times [50].

Chau [44] evaluated the effect of incorporating SCGs from Arabica and Robusta varieties at 2.0% and 4.0% levels on the physical properties of bread (crust, crumb color, specific volume, density, and texture). A chromameter was used to assess the crust and crumb color. Specific volume and density were measured using the AACC 10-05.01 rapeseed displacement technique [54]. Furthermore, texture parameters were determined using a texture analyzer [44]. The crumb of SCG-enriched bread showed a darker and redder color due to the high polyphenol and melanoidin content resulting from the Maillard reaction during coffee roasting [7,32]. Higher SCGs concentrations led to darker crusts and crumbs [44]. Arabica SCGs had no significant effect on specific volume or density, whereas Robusta SCGs reduced specific volume and increased density. This was attributed to the phenolics and dietary fiber in the SCGs competing with wheat flour proteins for water, which hindered gluten network formation and reduced gas retention during fermentation [57,58]. Bread hardness increased with SCGs incorporation, alongside a trend toward increased gumminess, attributed to the reduced yeast and dough enzymatic activity [59,60]. Cohesiveness and resilience decreased with SCGs addition, resulting in a crumbly texture. However, the differences between the SCG-enriched and control bread diminished over storage time [44], suggesting potential benefits in extending shelf life by delaying staling [61,62].

#### 3.2.3. Biscuits

Ali et al. [46] examined the impact of incorporating SCGs into the color characteristics of biscuits at concentrations of 2.0%, 4.0%, and 6.0%. A spectro-colorimeter was used to measure the biscuits’ color. They observed that with the increase in the amount of dark SCGs, the L* value indicated that the biscuits’ lightness decreased. The a* value, representing redness, increased with higher SCGs levels, while the b* value, indicating yellowness, decreased compared to the control. This contradicted expectations based on the Maillard reaction, where increased a* and b* values typically correlate with heightened redness and yellowness [63]. The discrepancy suggests that the brown hue from SCGs might be too dark to be visually perceived, complicating interpretation [64]. Therefore, these findings do not indicate the browning reaction’s progression during SCGs incorporation into biscuits [46].

#### 3.2.4. Cakes

Hussein et al. [31] investigated the physical properties of sponge cakes containing 2.0%, 4.0%, and 6.0% SCGs. A tristimulus colorimeter with the CIELAB color space was used to measure color factors. The farinogram characteristics of dough blends were assessed to measure rheological attributes according to AACC guidelines [65]. The volume, weight, and specific volume of samples were measured using the AACC technique [65]. A testing system equipped with a cylinder probe was used to evaluate texture parameters. The findings indicated that adding SCGs intensified browning in both crust and crumb compared to the control cakes. Initial nonenzymic browning led to increased redness (a*) and yellowness (b*). Regarding the rheological features of cake dough, adding SCGs increased water absorption and the dough development time. In addition, dough stability decreased with SCGs inclusion, indicating a weaker dough. The primary component of SCGs, fibers, significantly influences the rheological characteristics of the resulting sponge cake. Regarding baking quality, cakes containing SCGs showed an increase in specific volume and weight compared to the control. In terms of textural characteristics, cakes with SCGs had lower hardness but higher adhesiveness and lower gumminess and chewiness compared to the control [31].

Ahmed et al. [28] studied the impact of supplementing sponge cakes with SCG powder at 1.0%, 2.0%, and 3.0% levels. Color properties were analyzed using the Minolta calorimeter. These researchers reported that these additions did not significantly affect the cakes’ weight, height, and specific volume compared to the control sample. In terms of color properties, the crust’s brightness (L*) decreased in products with higher SCGs, except for sponge cakes with 1.0% SCGs. There was no notable change in redness (a*) across samples. Yellowness (b*) decreased significantly in all coffee-supplemented cakes. Additionally, the crumb’s brightness (L*) decreased in all samples, while the cake containing 1.0% SCGs showed no significant change in redness (a*). The increase in redness (a*) in other samples suggests the initiation of the Maillard reaction [66]. Yellowness (b*) remained unchanged across all SCG-supplemented cakes.

#### 3.2.5. Muffins

Severini et al. [48] evaluated the effect of adding SCGs at concentrations of 15.0% and 30.0% on the physical characteristics of muffins. A digit CAL SI caliper, colorimeter, and texture analyzer were used to assess the muffins’ dimensions, color, and texture profile, respectively. The results indicated no significant difference in the overall dimensions of muffins containing SCGs and the control treatment. However, there were notable differences in how the pores were distributed throughout each portion. In terms of color attributes, the integration of SCGs led to a reduction in color factors (L*, a*, b*). In terms of texture, adding SCGs to muffins did not cause significant changes in textural parameters such as hardness, springiness, cohesiveness, chewiness, and gumminess.

Benincá et al. [49] conducted a study investigating the impact of SCGs at various concentrations (1.0%, 16.0%, 31.0%, 46.0%, and 61.0%) on the color and texture of muffins. These researchers used a colorimeter and a texture analyzer, respectively, to check the color and texture attributes of the muffins. The addition of SCGs resulted in significant differences in color characteristics, with lower L* values found in muffins with higher concentrations of SCGs, which could be due to the Maillard reaction producing melanoidins. SCG-enriched muffins had lower a* and b* values than control muffins. Texture analysis revealed greater hardness in SCG-enriched muffins. However, parameters like elasticity, chewiness, and cohesiveness did not significantly differ between control and SCG-enriched muffins up to 61.0%, indicating that SCGs substitution for wheat flour did not alter the textural aspects. The study concluded that the addition of SCGs of up to 61.0% maintained the muffin structure while enhancing hardness, suggesting potential applications in product development.

#### 3.2.6. Pasta

Sugianto [19] investigated the cooking quality (optimal cooking time, water uptake, cooking loss, and swelling index), hardness, and pasta color, containing 4.0%, 8.0%, and 10.0% of SCGs. The optimal cooking time for pasta was measured by boiling 10 g of pasta in 100 mL of water. Starting at 5 min, a piece was removed every minute and compressed between glass plates to check for a white core. The time was recorded when no white core remained. Fifteen grams of pasta were placed in a stainless-steel strainer and submerged in 300 mL of boiling water for 9 min to assess the cooking quality, including water uptake, cooking loss, swelling index, and hardness. After cooking, the pasta was rinsed under cold water, shaken to remove excess water, and left to drain for 5 min before weighing. To measure the swelling index, cooked pasta was dried at 105 °C until a constant weight was reached [67]. The swelling index was calculated as the ratio of the cooked product weight to its dried weight. In terms of the optimal cooking time, pasta containing SCGs tended to break more easily than commercial pasta when cooked, suggesting that the optimal cooking time for SCG-containing pasta might be lower than what was instructed by the pasta manufacturer due to the fiber weakening the gluten matrix [68]. Pasta with SCGs showed a higher water uptake compared to the control, possibly due to the differences in drying techniques affecting the moisture content and higher fiber content disrupting the protein–starch matrix. SCG incorporation reduced the pasta swelling index [19], likely due to the presence of fiber and protein forming cohesive networks that impede water flow and starch gelatinization [66,69,70]. Uncooked pasta with SCGs, particularly at higher concentrations, exhibited greater hardness, indicating a potential impact of SCGs on pasta texture [19]. Cooked pasta with SCGs retained its shape and exhibited color differences, with SCGs contributing to darker coloration [19].

The findings indicate that including SCGs in baked goods affects various physical characteristics, including texture and color [19,28,29,36,44,46,49,50]. SCGs consistently improve features in cookies, bread, and cakes, primarily through the Maillard reaction, which darkens the color and alters the textural properties [7,29,31,32,44,50]. A common trend across products is the reduction in lightness and an increase in hardness, although specific effects vary by formulation [18,29,44,46,49]. For instance, SCGs increase cookie fracturability [29] but may decrease bread cohesiveness [50], illustrating product-specific differences. On the other hand, the impact of SCGs on the texture, color, and cooking quality of food products—while differing based on the specific product and the concentration of SCGs—underscores their potential to contribute to the development of healthier and more innovative food options [44,49].

### 3.3. Bakery Products’ Microbiological Properties

Desai et al. [33] investigated the impact of adding UGCSF and RGCSF flour (3.0%, 6.0%, 10.0%, and 12.0%) on the microbiological characteristics of cookies. The study demonstrated that the UGCSF had a microbial load of less than 80 CFU/g for endospores, while yeast, mold, and aerobic bacteria were undetected. The absence of mold in UGCSF undoubtedly lowers the possibility of ochratoxin exposure. It is evident that the UGCSF has outstanding microbiological quality and, as a result, has the potential to be a food ingredient that is safe and rich in nutraceuticals. RGCSF had less than 10 CFU/g. The microbial burden was safe across all cookies examined for this research.

Sharma et al. [36] evaluated the probiotic features of cookies supplemented with 5.0, 7.0, and 10.0% RSCGs and SCGR. The results showed that both cookie types had a similar probiotic viable cell count.

Martinez Saez et al. [30] counted aerobic microorganisms, endospores, mold, and yeast in biscuits enriched with 3.50%, 3.64%, 3.77%, 3.94%, 4.24%, and 4.40% of SCGs. Findings showed that adding SCGs did not influence the number of microorganisms in the biscuit formulation and complied with the national microbiological requirements for biscuits (RD 1124/82, 1982). Also, the total aerobic microorganisms were reported to be 104 CFU/g, while endospores, mold, and yeasts were lower than 102 CFU/g for the biscuit containing 4.24% SCGs.

Ahmed et al. [28] investigated the microbial properties of sponge cakes containing SCGs at 1.0%, 2.0%, and 3.0% stored for 0, 7, and 14 days. The total bacterial count, mold, and yeast number elevated significantly in the control cake and in cakes supplemented with used SCGs at 7 and 14 days of storage compared to cakes stored for 0 days. However, sponge cakes supplemented with varying levels of SCGs demonstrated a noticeable reduction in the total bacterial count, total mold, and yeast compared to sponge cake without SCGs during each storage period, indicating the positive impact of SCGs on the microbial quality of the cakes.

Incorporating SCGs into baked goods does not compromise microbiological safety [28,33]. The studies indicate that SCGs can be safely utilized as a nutritious ingredient in various baked goods, ensuring their quality and safety for human consumption [28,30,33,36].

### 3.4. Bakery Products’ Sensory Properties

#### 3.4.1. Cookies

Aguilar-Raymundo et al. [27] used a nine-point hedonic scale to rate the sensory characteristics of cookies containing concentrations of 10.0%, 17.5%, and 25.0% SCGs. In addition, they studied the sensory parameters (hardness, granularity, flavor, and taste) of cookies using a Just-About-Right scale (JAR) method. The findings indicated that cookies with 17.5% SCGs were the favorites among consumers, followed by those with 10.0% SCGs. However, no significant difference in overall liking was noted across the formulations. While over 56% of participants found the cookies’ coffee taste acceptable, most found the hardness low, and about 50% thought the coffee aroma was insufficient. The study suggests that SCGs concentrations between 17.5% and 25.0% might be optimal, as higher levels of SCGs could make the coffee taste overwhelming. Overall, cookies with SCGs were enjoyed, but those with 17.5% SCGs were preferred in terms of sensory attributes like hardness, granularity, flavor, and taste.

In another study by Sérioa et al. [35], the effect of adding 1.0%, 2.0%, and 3.0% of the SCG extract on the sensory properties of cookies was tested. The participants were reported to prefer samples with fewer incorporated extracts.

Azuan et al. [29] evaluated the sensory parameters of cookies supplemented with 0.27%, 0.53%, 0.80%, 1.07%, and 1.33% SCG extracts from Arabica coffee involving 40 untrained assessors using a nine-point hedonic scale. There was no discernible difference in the scores for the appearance of the SCG extract-supplemented and the control samples. Nonetheless, the control sample indicated the highest appearance score, followed by samples containing 0.53%, 0.80%, 1.07%, 1.33%, and 0.27% SCG extracts. The sample supplemented with 0.53% SCG extract demonstrated the highest color score in lightness, followed by samples containing 0.80%, 1.33%, and 1.07% SCG extracts, the control, and 0.27% SCG extract.

Desai et al. [33] assessed the effects of UGCSF and RGCSF in cookies at amounts of 3.0%, 6.0%, 10.0%, and 12.0% using a seven-point scale. They reported that cookies containing 10.0% and 12.0% RGCSF had more favorable sensory properties in terms of color, appearance, aroma, taste, crispiness, and overall acceptability.

Meerasri and Sothornvit [34] examined the impact of the inclusion of 10.0%, 20.0%, and 30.0% extracted oil from SCGs on the sensory attributes of cookies. These researchers used a nine-point hedonic scale to examine these attributes. No significant differences were found for color, hardness, crumbliness, and odor scores between cookies that contained SCG oil and thee control. On the other hand, the cookies with 20.0% or 30.0% SCG oil received lower flavor rating scores. The higher contents of SCG oil caused the cookies to smell like cooked coffee oil and impacted the mouthfeel when consumed.

Sharma et al. [36] investigated the sensory features of cookies supplemented with 5.0%, 7.0%, and 10.0% SCG residue (RSCG) using a nine-point hedonic scale. Cookies supplemented with 7.0% RSCG had the highest general acceptance rate in terms of texture and taste. At the same time, an enhancement in RSCG content gave the cookies an extra bitter taste and negatively impacted their texture and overall acceptance. SCG oil has a strong smoky smell associated with bitterness and astringency; thus, it cannot be utilized to bake cookies.

Trà et al. [18] studied the sensory characteristics of cookies enriched with 0.05%, 0.10%, 0.15%, 0.20%, and 0.25% of SCGs using a nine-point hedonic scale. They reported that the general acceptance of the SCG-enriched cookies remained unchanged when the SCGs ratio increased from 0.05% to 0.20%. However, the sensory score of cookies with 0.25% SCG dropped, which most likely resulted from the product’s significantly greater hardness.

Oliveira Batista et al. [32] proposed developing gluten-free cookies with SCGs (10.0%, 20.0%, and 30.0%). These researchers used a nine-point hedonic scale to evaluate sensory properties. The flavor and general acceptability of the cookies declined when higher concentrations of SCGs (30.0%) were used in the cookies’ formula. There were no variations in the assessed formulations’ acceptance of texture and appearance. Scores of samples enriched with SCGs ranged from 5.95 to 6.87 across the analyzed qualities, indicating that consumers found some samples to be neither very appealing nor very objectionable. The cookies’ acceptability parameters revealed that the formulations containing 10.0% and 20.0% SCGs were acceptable and suitable for commercialization.

Koay et al. [7] evaluated the effect of SCGs at levels of 2.0%, 4.0%, 6.0%, 8.0%, and 10.0% on the sensory parameters of cookies using a nine-point hedonic scale. In terms of color, the control group scored highest (7.15), with the acceptability decreasing as the SCGs levels increased due to the darker color of SCGs. Interestingly, the 10.0% SCGs shortbread ranked second in color preference, likely because panelists found the darker hue more prosperous. In terms of aroma, SCGs levels significantly influenced aroma, with 10.0% of SCGs receiving higher scores. However, there was no significant difference between the 6.0% and 8.0% SCGs shortbreads, likely due to the loss of volatile aromatics during coffee brewing. In terms of taste, no significant difference was observed in the taste of 2.0% to 8.0% of SCGs shortbreads. However, the control sample outperformed the 10.0% SCG shortbread, possibly due to the presence of bitter compounds like caffeine. In terms of hardness, the 10.0% SCGs shortbread was comparable to the control in hardness, while lower SCG concentrations showed significant differences from the control. Regarding fracturability and oiliness, SCGs incorporation did not affect the shortbread’s crumbliness. The oiliness of the 10.0% SCGs inclusion in shortbread was comparable to the control in acceptability. The control sample scored highest in general acceptability, followed by 10.0% SCGs shortbread. Thus, SCGs can be incorporated into shortbread without negatively impacting sensory qualities.

#### 3.4.2. Bread

Daniel et al. [42] investigated bread’s sensory parameters (color, taste, aroma, and texture) enriched with 2.0%, 4.0%, 6.0%, 8.0%, and 10.0% SCGs using a nine-point hedonic scale. There were noticeable color variations between the control and SCG-enriched breads. The color acceptance of bread decreased as the amount of added SCGs increased. The aromas of white and bread with 6.0%, 8.0%, and 10.0% SCGs incorporations did not change significantly. The acceptability of the texture of the bread diminished with the addition of SCGs.

Rwubatse et al. [50] tested the sensory properties of whole wheat bread formulated with 4.0% SCGs using a seven-point hedonic scale. The findings indicated that customers were satisfied with the aroma and taste of whole wheat bread containing SCGs.

#### 3.4.3. Biscuit

Martínez Saez et al. [30] studied the sensory characteristics of biscuits containing 3.50%, 3.64%, 3.77%, 3.94%, 4.24%, and 4.40% SCGs compared to commercial biscuits using a seven-point hedonic scale. The findings showed that biscuits containing 3.50%, 3.64%, and 4.24% of SCGs had the highest scores for color. Regarding the texture and taste, no significant difference was reported between biscuits containing 3.64% and 4.24% SCGs with commercial biscuits. Also, the overall acceptance was similar for biscuits, with 3.50%, 3.64%, and 4.24% SCGs compared to commercial biscuits.

Ali et al. [46] evaluated the effect of SCGs (2.0%, 4.0%, and 6.0%) on sensory parameters (aroma, taste, color, texture, and overall acceptance) of biscuits. The authors stated that no significant differences were found in the aroma and taste between biscuits containing 2.0% and 4.0% SCGs and the control. However, adding 6.0% SCGs caused a significant diminish in respected characteristics. In terms of color, a significant difference was observed between the samples containing SCGs and the control sample, and with the enhancement in the level of SCGs, the color score diminished. On the other hand, no substantial difference was observed between the samples containing SCGs and the control sample.

#### 3.4.4. Cakes

Hussein et al. [31] examined the sensory parameters (color, taste, smell, texture, appearance, and general acceptability) of sponge cakes enriched with 2.0%, 4.0%, and 6.0% SCGs. The findings showed a noticeable difference between the sponge cakes enriched with SCGs and the control sponge cake. The color, taste, smell, texture, appearance, and general acceptability of sponge cake incorporated with 2.0% and 4.0% SCGs were significantly higher than those of the samples with 6.0% SCGs. Reduced glycemic sugars significantly influence the acceptability of smell and taste, presumably due to their roasted, sweet, and caramel flavors, which are regarded as the primary and most favorable sensory characteristics. Notably, the texture of samples containing SCGs seems to be almost equal to or even better than that of the control group. This could be attributed to the high emulsifying activity and emulsion stability of SCGs.

Ahmed et al. [28] investigated the sensory parameters (appearance, crust color, crumb color, texture, taste, smell, and overall) of sponge cakes supplemented with 1.0%, 2.0%, and 3.0% SCGs using a ten-point numeric scale. They stated that all the sensory parameters decreased significantly in all baked cakes that contained SCGs.

#### 3.4.5. Muffins

Severini et al. [48] studied the sensory characteristics, including appearance, color, odor, off-odor, taste, softness, and overall acceptance, of muffins enriched with 5.25% and 10.50% SCGs using a five-point hedonic scale. In terms of general acceptance, appearance, and color, samples containing 10.50% SCGs scored higher than those containing 5.25% SCGs. There was no difference in the odor, taste, and softness of coffee samples, while the sample containing 10.50% SCGs had more off-odor than other samples. Overall, these researchers reported that the sensory characteristics of muffins gained higher scores (3.5) than average on a five-point scale, which indicates a good level of acceptance among the consumers.

The sensory assessment of baked goods enhanced with SCGs demonstrates a variety of results among cookies, bread, biscuits, cakes, and muffins [27,28,30,35,48,50]. Formulations with intermediate SCGs concentrations (about 10–20%) are frequently chosen in cookie studies due to their well-balanced flavor, color, and texture [27,32,33]. Nevertheless, higher SCGs levels may cause bitterness and strong coffee flavors, which would have a detrimental effect on acceptance [36]. Regarding bread, enhanced SCGs levels tend to diminish color acceptability, but the aroma remains largely unchanged [42]. Whole wheat bread containing 4% SCGs showed good acceptance in terms of taste and aroma [50]. Similarly to commercial biscuits, biscuits with SCGs ranging from 3.5% to 4.4% scored highest in sensory measures, suggesting that moderate SCGs additions have no discernible effect on texture or flavor [30]. Low amounts of SCGs (2–4%) improve the texture and flavor of cakes by imparting roasted and caramel-like characteristics. However, the sensory appeal of cakes with 6% SCGs decreases [31]. Finally, even though higher SCGs concentrations resulted in increased off-odor, muffins enhanced with 5.25% or 10.5%, SCGs demonstrated widespread acceptance [48]. Moderate amounts of SCGs can be added to bakery goods to improve sensory aspects without compromising taste or texture, making them acceptable to consumers.

## 4. Study Limitations

Analyses were conducted on the chemical, physical, microbiological, and sensory characteristics of baked goods (cookies, bread, biscuits, cakes, muffins, and pasta) that contained SCGs. The following problems were identified in various papers:

In some studies, the variety of coffee was not specified [7,27,31,35,36,46], and a few studies checked the microbiological properties of the baked goods [28,30,33,36]. Only one study was performed on pasta [19]. Some studies used small sample sizes or lacked diversity in participants or experimental conditions, potentially limiting the robustness and applicability of their results [28,38,47,50]. Some studies were relatively short and only provided a snapshot of the effects of SCGs incorporation [30,35,47]. Different storage conditions in studies affect the stability and sensory characteristics of cooked products containing SCGs [7,27,28,34,35,47,50]. Various processing methods used in different studies, such as differences in mixing, fermentation, and baking parameters, affected the final quality characteristics of baked products [7,29,33,34,50]. Differences in sensory evaluation methods, such as panel selection, training, and evaluation criteria, led to conflicting findings regarding consumer acceptance and preference [28,29,31,48,50]. The comparison of chemical, physical, microbiological, and sensory properties was also influenced in certain research reports by the standardization of the studies.

## 5. Conclusions

Incorporating spent coffee grounds (SCGs) into bakery products and pasta presents an opportunity to enhance their nutritional and sensory qualities. The chemical changes induced by SCGs’ inclusion contribute to increased protein, fat, fiber, ash, and antioxidant content, thereby improving the overall nutritional value of baked goods. Furthermore, SCGs can positively impact the physical properties of bakery items, although these effects depend on factors such as SCGs’ concentration, coffee variety, and processing methods.

Importantly, studies have shown that incorporating SCGs into bakery products does not compromise their microbial safety, ensuring the quality and safety of these goods for human consumption. To optimize the sensory properties of baked goods, careful consideration must be given to factors including SCGs’ concentration and the type of baked product. Sensory properties encompass how food is perceived by the senses of taste, aroma, appearance, and mouthfeel, which are crucial for consumer acceptance. Optimizing these properties requires finding the right balance of SCGs in the recipe. Too much SCGs can overpower the flavor, while too little may not sufficiently enhance the taste or aroma. Different types of baked goods respond to SCGs uniquely due to their distinct structures and textures. For example, cookies, which have lower moisture and a higher density than bread or cakes, may benefit more from adding SCGs in terms of flavor and aroma enhancement. Moreover, storage conditions, such as temperature and humidity, are critical in maintaining sensory quality over time. SCGs can affect the shelf life, flavor retention, and freshness of baked goods, which is essential for ensuring continued consumer enjoyment even after extended storage.

From an economic perspective, upcycling SCGs supports a circular economy model by diverting coffee waste from landfills, thus contributing to significant waste reduction and minimizing the food industry’s environmental footprint.

Compared to other food supply chain applications, such as animal feed, biofuel production, and composting, the use of SCGs in bakery products adds value through human nutrition while still contributing to sustainability goals. This highlights the versatility of SCGs as a functional ingredient, offering benefits beyond other common applications.

In conclusion, utilizing SCGs in bakery products holds substantial promise for creating innovative, nutritious, economically viable, and sustainable food formulations. Further research is needed to refine optimal formulations and processing techniques to fully harness the potential of SCGs incorporation into bakery applications, ensuring maximum benefits in terms of nutrition, sensory quality, and environmental impact.

## Data Availability

No new data were created or analyzed in this study. Data sharing is not applicable to this article.
